# Revolutionizing Tuberculosis Management With Clustered Regularly Interspaced Short Palindromic Repeats (CRISPR)-Cas Technology: A Comprehensive Literature Review

**DOI:** 10.7759/cureus.71697

**Published:** 2024-10-17

**Authors:** Achal Shetty, Hamida Kwas, Hayfa Rajhi, Harish Rangareddy, Jessica Fryer

**Affiliations:** 1 Community Medicine, Father Muller Medical College, Mangalore, IND; 2 Pulmonology, University of Sfax, Faculty of Medicine of Sfax, Gabès University Hospital, Gabès, TUN; 3 Analysis Laboratory Research, University Hospital of Gabès, Gabès, TUN; 4 Biochemistry, Haveri Institute of Medical Sciences, Haveri, IND; 5 Internal Medicine, Meditrial, KwaZulu-Natal, ZAF

**Keywords:** crispr/cas9 gene editing, crispr-cas system (clustered regularly interspaced short palindromic repeats)-crispr associated system, gene editing, multi-drug resistant bacteria, tuberculosis

## Abstract

Clustered regularly interspaced short palindromic repeats (CRISPR)-Cas systems have gained attention for their revolutionary potential in tuberculosis (TB) management, providing a novel approach to both diagnostics and treatment. This technology, renowned for its ability to accurately target and modify genetic material, offers a promising solution to the limitations of current TB diagnostic methods, which often rely on time-consuming culture techniques or polymerase chain reaction (PCR)-based assays. One of the key advantages of CRISPR-Cas systems is their high specificity and sensitivity, making them well-suited for detecting *Mycobacterium tuberculosis*, even in low-bacterial-load samples. Techniques such as CRISPR-Cas12 and Cas13 have been employed for rapid detection, utilizing their trans-cleavage activity to produce a fluorescent signal upon recognition of the TB genome. Furthermore, these methods often use isothermal amplification techniques like recombinase polymerase amplification (RPA) or loop-mediated isothermal amplification (LAMP), which require less equipment compared to traditional PCR.

Beyond diagnostics, CRISPR-Cas technologies show promise in studying TB resistance mechanisms and potentially treating drug-resistant strains. Genome-editing capabilities enable researchers to manipulate the *M. tuberculosis* genome, investigating genes linked to virulence or antibiotic resistance. Although challenges such as the development of multiplexed CRISPR assays for detecting multiple mutations simultaneously remain, advancements continue to improve the technology’s practicality for clinical use. Incorporating CRISPR into TB management could enhance early detection, inform personalized treatment, and potentially contribute to developing more effective therapies, especially in regions where TB remains a significant public health threat.

## Introduction and background

Genome editing involves modifying genomic DNA at specific target sites in various cell types and organisms. This includes the insertion, deletion, and replacement of DNA, which can result in the inactivation of target genes, acquisition of novel genetic traits, or correction of pathogenic gene mutations [1]. Scientists have long sought effective methods to manipulate DNA and RNA to modify genes and their regulation. Genetic perturbation allows researchers to investigate gene function or rectify mutations, but it is often difficult due to the technical hurdle of targeting nucleic acids with precision. While targeted gene editing has been accomplished by inducing double-stranded DNA (dsDNA) breaks in eukaryotic chromosomes, these efforts have traditionally relied on complex techniques that require engineering protein-DNA recognition systems [2].

Genome editing through double-stranded breaks (DSBs) has revolutionized genetic manipulation. Programmable nucleases are employed to induce sequence-specific DSBs in genomic DNA. Without a repair template, the cell primarily repairs the break through non-homologous end joining (NHEJ), which often introduces insertions or deletions (indels) at the editing site. However, if a separate DNA template with sequences homologous to the regions flanking the DSB is provided, homologous-directed repair (HDR) can incorporate the desired template into the genome. Technologies like zinc finger nucleases (ZFNs), transcription activator-like effector nucleases (TALENs), and clustered regularly interspaced short palindromic repeats (CRISPR)-based nucleases have been instrumental in creating programmable, sequence-specific DSBs. Unlike ZFNs and TALENs, which require engineering new DNA-binding, the CRISPR-associated protein (Cas) systems have simplified the reprogramming process [3]. Due to their affordability, efficiency, and repeatability, CRISPR-Cas systems have become the most used genome editing technology worldwide [3,4].

The CRISPR-Cas system as an adaptive immune defense in prokaryotes protects against subsequent phage infections by integrating viral DNA into the host genome for future recognition and destruction. This viral DNA, flanked by repetitive sequences called direct repeats, is located near genes encoding Cas proteins. The system has been repurposed for genome editing by directing reprogrammed endonucleases to specific target genes, enabling precise modifications. CRISPR allows for reprogrammable alterations in both DNA and RNA making it a powerful tool for sequence-specific genome editing. The Cas has found broad applications across various fields, including agriculture, therapeutics, infectious disease research, food industry, and bioenergy [5]. In recent years, researchers have made significant strides in improving the efficiency, specificity, and versatility of CRISPR-Cas systems. This review will discuss the role of CRISPR-Cas systems in tuberculosis (TB) management, highlighting new Cas enzymes, base editing, gene regulation, and CRISPR diagnostics.

## Review

Tuberculosis: epidemiology and impact

TB remains one of the deadliest infectious diseases worldwide, caused by the bacterium *Mycobacterium tuberculosis* (MTB). According to the World Health Organization (WHO), approximately 10 million people developed TB and 1.3 million people died from the disease in 2022 globally [6]. TB is particularly prevalent in low- and middle-income countries (LMICs), with a significant impact on public health and economic stability [7]. Treating TB requires a combination of multiple drugs administered over several months, posing significant challenges for both patients and healthcare systems, particularly in resource-limited settings. This is exacerbated by the growing prevalence of drug-resistant TB, which requires even longer, more costly, and less tolerable treatment regimens. Diagnosis in LMICs typically relies on microscopic examination of stained sputum smears from suspected TB patients. However, smear microscopy detects only 50-60% of cases (smear-positive), highlighting the urgent need for more sensitive diagnostic methods and tools to better identify drug resistance [8].

Pathogenesis and transmission

TB is primarily transmitted through airborne particles when a person with active pulmonary TB coughs, sneezes, or speaks [9]. Once inhaled, the bacteria can infect the lungs, where they are ingested by alveolar macrophages. MTB can survive and replicate within these immune cells while evading the host's immune response. The infection can remain latent for years before reactivating and causing active disease, particularly in individuals with weakened immune systems [10].

MTB has evolved several mechanisms to evade host immune defenses, one of the most critical being its ability to inhibit the fusion of lysosomes with phagosomes, a process essential for bacterial degradation. This is mediated by several MTB-derived factors, including the secretion of protein kinase G (PknG), which prevents the maturation of the phagosome, and suppression of nuclear factor-κB, which is crucial for initiating immune responses [11-13].

Another important evasion mechanism involves the manipulation of phosphatidylinositol 3-phosphate (PI3P), a key lipid that regulates phagosomal maturation. MTB reduces PI3P biosynthesis while also increasing its hydrolysis, thereby blocking the recruitment of proteins necessary for the fusion with lysosomes [14,15]. These strategies collectively allow MTB to establish a niche within macrophages, contributing to its survival and the progression of TB.

Current challenges

Despite advancements in molecular TB diagnostics, several challenges persist. Current nucleic acid amplification tests (NAATs) demonstrate sensitivity and specificity exceeding 90% and 95% for respiratory samples, outperforming traditional microbiological methods. However, sensitivity remains lower in paucibacillary cases, prevalent in children and individuals co-infected with human immunodeficiency virus (HIV) [16,17]. The next-generation GeneXpert Ultra test has a sensitivity of 73% and specificity of 97% in sputum samples from children with suspected pulmonary TB, and 88% sensitivity and 95% specificity in adults living with HIV. Sensitivity is even lower in non-respiratory samples, such as cerebrospinal and pleural fluids, ranging from 50% to 70% [18].

The standard treatment for TB involves a combination of antibiotics over the course of six to nine months [8]. However, the emergence of multidrug-resistant TB (MDR-TB) and extensively drug-resistant TB (XDR-TB) has posed significant challenges to TB control. These forms of TB are resistant to the most effective first-line and second-line antibiotics, necessitating the development of new therapeutic strategies [19].

Mechanism of CRISPR-Cas

**Figure 1:**
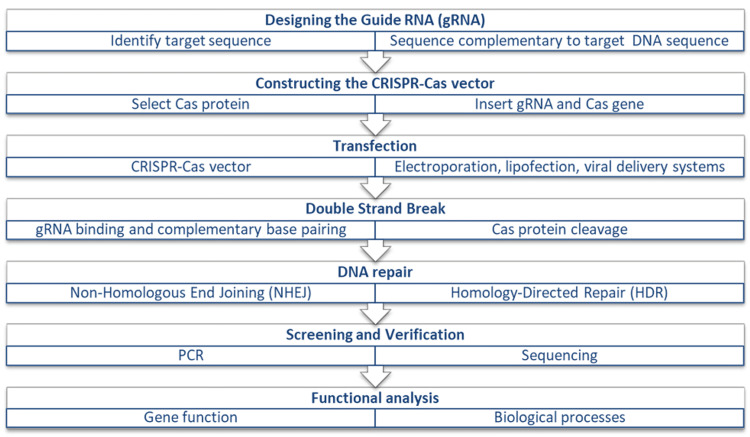
General steps of gene editing using CRISPR-Cas. CRISPR: clustered regularly interspaced short palindromic repeats; Cas: CRISPR-associated protein; PCR: polymerase chain reaction. Source: The image is created by Harish Rangareddy and is marked with a CC0 1.0 Universal license (https://creativecommons.org/publicdomain/zero/1.0/).

Figure 1 shows the key stages involved in CRISPR-Cas gene editing, from identifying the target DNA sequence to making precise genetic modifications. This process outlines how the CRISPR-Cas system facilitates targeted alterations in the genome with high accuracy.

The CRISPR-Cas9 system comprises two essential components: guide RNA (gRNA) and CRISPR-associated protein 9 (Cas9). The mechanism of CRISPR-Cas9 genome editing involves three steps: recognition, cleavage, and repair. The designed single-guide RNA (sgRNA) recognizes the target sequence in the gene of interest through complementary base pairing. The Cas9 nuclease makes double-stranded breaks at a site three base pairs upstream of the protospacer adjacent motif (PAM). The resulting double-stranded break is then repaired by either NHEJ or homology-directed repair (HDR) cellular mechanisms [3].

Applications in TB management

CRISPR Therapeutics

Recent discoveries have identified new Cas enzymes that enhance the functionality of CRISPR-Cas systems. These enzymes can target different DNA sequences and RNA molecules, perform multiplex genome editing, or edit large DNA fragments [20]. MTB was long believed to have a conserved CRISPR locus with no impact on bacterial physiology, as its diversity seemed unrelated to virulence [21]. However, recent findings suggest that MTB strains with a complete set of Cas proteins can degrade plasmid DNA sequences homologous to CRISPR spacers, challenging this view [22].

Most *Mycobacterium* species have a single type IIIa CRISPR-Cas system [23]. An exception is *Mycobacterium canettii*, an environmental species occasionally infecting humans, which may harbor diverse class 1 CRISPR-Cas types [24]. These systems were likely acquired via horizontal gene transfer from other bacteria [25].

Engineering new Cas enzymes to enhance their specificity, activity, and delivery to target cells is crucial for TB management. These enzymes can be tailored to target the MTB genome, potentially disrupting genes essential for its survival and virulence [25].

Cas9 leads to MTB death by introducing targeted double-strand breaks (DSBs) in the bacterial DNA. These DSBs occur at specific sites in the genome, guided by a sgRNA that directs Cas9 to the corresponding DNA sequence. In MTB, the DNA repair systems, such as NHEJ and homologous directed recombination, attempt to repair the Cas9-induced DSBs [26,27]. However, when these repair mechanisms are overwhelmed or fail, the accumulation of unrepaired or incorrectly repaired DSBs leads to genomic instability, loss of essential genetic information, and eventual cell death. The lethality of Cas9 is particularly pronounced when no homologous repair template is available, as the resulting random insertions or deletions (indels) from NHEJ can disrupt crucial genes or regulatory elements, leading to bacterial death.

The rapid advancements in CRISPR-Cas-based genome editing technologies have introduced several sophisticated techniques for genetic manipulation in mycobacteria. These approaches, each designed to overcome specific technical challenges, hold promise for functional genomics and therapeutic interventions.

CRISPR interference (CRISPRi) employs a mutant Cas9 (dCas9) from *Staphylococcus* that lacks endonuclease activity, allowing for precise regulation of gene expression by interfering with target gene transcription in *Mycobacterium smegmatis* and MTB [28,29]. CRISPR-assisted-recombineering utilizes Cas12a (class 2, type Va) and involves co-transfection with double- or single-stranded DNA (ssDNA) carrying desired mutations, facilitating the generation of point mutations and indels in *Mycobacterium* *smegmatis.* This technique is enhanced by coupling with mycobacteriophage Che9c to improve recombination efficiency [30]. CRISPR-FnCpf1-assisted-NHEJ, also utilizing Cas12a, combines overexpression of NHEJ proteins with inhibition of RecA-dependent homologous recombination (HR) to create deletions and double mutations. This approach has been effectively applied to *Mycobacterium* *marinum, Mycobacterium smegmatis,* and MTB [31,32].

CRISPR1-Cas9 utilizes a modified version of Cas9, Sth1dCas9, with restored nuclease activity to generate insertions or deletions (indels) by inducing DSBs near the PAM sequence. This method has been successfully applied in *Mycobacterium* *marinum, Mycobacterium* *smegmatis*, and MTB [33]. The type III CRISPR system, employing endogenous Csm/Cmr complexes, facilitates gene knock-ins, knockouts, and RNA interference through the cleavage of RNA and ssDNA. This system, guided by CRISPR RNA (gRNA) and HDR templates, enables precise gene editing in MTB [34].

Base editing is a technique that enables precise changes to individual nucleotides in DNA without introducing double-stranded breaks. This is accomplished by fusing a Cas enzyme with a DNA-modifying enzyme that can change one nucleotide into another. In TB management, base editing can be used to introduce specific mutations in the MTB genome that attenuate its virulence or enhance the host's immune response. This technique has enormous potential for developing novel TB therapies by precisely targeting and modifying critical MTB genes. The base editing system leverages a modified Cas9 (nCas9) fused with APOBEC1 and uracil-DNA glycosylase inhibitor (UGI) to convert single bases (G: C to A: T) without altering the surrounding sequence, offering a site-directed mutagenesis approach with minimal off-target effects [35].

Lastly, the CRISPR-guided DNA polymerase system (CAMPER) couples nCas9 with an error-prone DNA polymerase to induce random substitutions during nick translation, further reducing off-target effects while enabling site-specific mutagenesis in *Mycobacterium​​​​​​​ smegmatis* and MTB​​​​​​​ [36]. These cutting-edge techniques collectively enhance the precision and efficiency of genetic manipulation in mycobacterial species. Thus, Cas9 can be exploited as a bactericidal tool by precisely targeting essential genes in MTB, disrupting their function and leading to cell death.

CRISPR Diagnostics

**Figure 2:**
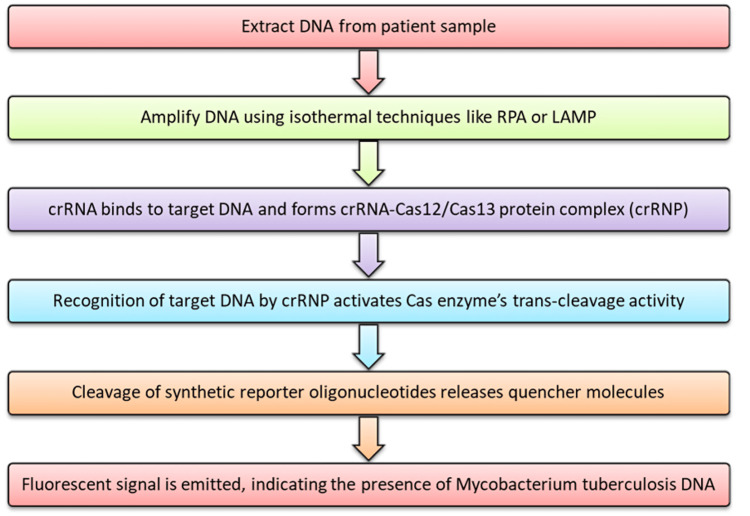
Steps in the diagnosis of TB using CRISPR. CRISPR: clustered regularly interspaced short palindromic repeats; Cas: CRISPR-associated protein; TB: tuberculosis; RPA: recombinase polymerase amplification; LAMP: loop-mediated isothermal amplification; crRNA: CRISPR RNA; crRNP: crRNA-Cas12/Cas13 protein complex. Source: The image is created by Harish Rangareddy and is marked with a CC0 1.0 Universal license (https://creativecommons.org/publicdomain/zero/1.0/).

CRISPR-based diagnostics are highly sensitive, specific, fast, and cost-effective, making them ideal for point-of-care diagnostics, especially in resource-limited settings. The general process, as depicted in Figure 2, begins with the extraction of DNA from the patient sample.

After extraction, the DNA is subjected to nucleotide amplification using isothermal techniques such as recombinase polymerase amplification (RPA) or loop-mediated isothermal amplification (LAMP). These methods are advantageous because they require minimal equipment compared to traditional polymerase chain reaction (PCR) [37,38].

The use of RPA or LAMP significantly streamlines the process, allowing rapid and highly sensitive detection of MTB, making CRISPR-Cas a promising diagnostic approach for use in resource-limited settings, where traditional PCR equipment may not be readily available [39,40]. LAMP, an isothermal amplification method, operates at a constant temperature, making it simpler and more accessible compared to traditional PCR, which requires thermal cycling. The advantage of LAMP lies in its speed and efficiency, often completing amplification within 30-60 minutes. When coupled with Cas12b, a CRISPR-associated nuclease with thermostable properties, the system becomes even more efficient for diagnostic purposes as one-pot detection is possible [40].

One-pot detection is advantageous because it (a) simplifies the workflow, (b) reduces the time required for diagnostics, (c) minimizes the need for advanced equipment, making it suitable for point-of-care or low-resource settings, and (d) reduces the risk of cross-contamination between multiple reaction steps [38].

Once amplification is complete, the CRISPR RNA (crRNA) associates with the Cas12 or Cas13 enzyme to form a crRNA-protein (crRNP) complex. Upon recognition of the specific target MTB DNA, the crRNP activates the Cas enzyme’s trans-cleavage activity, which not only cleaves the target DNA but also cuts synthetic reporter oligonucleotides that are quenched by attached molecules. This cleavage results in the release of the quencher molecule, thereby emitting a fluorescent signal. This fluorescent signal indicates the presence of MTB DNA in the sample [41,42]. The high specificity of this system is driven by the precise recognition of target sequences by the crRNA, ensuring minimal off-target effects. This feature is crucial in distinguishing *M. tuberculosis* from other mycobacterial species and pathogens, thereby enhancing diagnostic accuracy [41].

Recent advances in CRISPR-Cas systems

Prime Editing

Prime editing is a new CRISPR-based genome editing technique that allows for the precise editing of specific DNA sequences without cutting the DNA double-strand, reducing the risk of unintended mutations. This technique uses a modified Cas enzyme fused with an engineered reverse transcriptase to insert, delete, or replace specific nucleotides in the genome [43]. In TB management, prime editing can be used to introduce precise mutations in MTB genes or host genes involved in the immune response, providing new avenues for therapeutic intervention.

CRISPR-Cas Multiplexed Screens

CRISPR-Cas multiplexed screens are powerful tools for identifying genes involved in specific biological processes or diseases. Recent developments allow researchers to target multiple genes simultaneously. One of the main challenges in using multiplexed CRISPR systems for TB diagnosis lies in the non-specific trans-cleavage activity of the CRISPR-Cas system, which complicates the design of tests that can target multiple mutations simultaneously. This poses a significant limitation, particularly in detecting drug resistance, as the sensitivity of CRISPR-based assays is reliant on the ability to test multiple mutations [44]. Current PCR-based diagnostic kits, for instance, typically focus on detecting four to five mutations associated with rifampicin resistance. While multiplexing strategies for CRISPR diagnostics are under development, they may involve increased costs or greater assay complexity, potentially reducing the benefits that CRISPR-based testing offers [37].

## Conclusions

CRISPR-Cas systems continue to expand the frontiers of biotechnology, offering new horizons in TB management. The development of new Cas enzymes, base editing, gene regulation, and CRISPR diagnostics provides exciting possibilities for treating TB and advancing precision medicine. As researchers continue to innovate and refine CRISPR-Cas systems, the future of TB management looks promising. CRISPR technology could potentially be used to edit somatic cells of individuals with TB or even modify the germline to prevent TB transmission. These advancements highlight the transformative potential of CRISPR-Cas systems in revolutionizing TB treatment and control.
